# Pulmonary Embolism Complicated by Acute Limb Ischemia Managed by Surgical Pulmonary Embolectomy

**DOI:** 10.7759/cureus.14598

**Published:** 2021-04-20

**Authors:** Alexandru Marginean, Jeremiah F Haines, R. Anthony Perez-Tamayo, Carlos Bechara, Amir Darki

**Affiliations:** 1 Department of Cardiology, Loyola University Medical Center, Maywood, USA; 2 Department of Medicine, Loyola University Medical Center, Maywood, USA; 3 Department of Cardiovascular Surgery, Loyola University Medical Center, Maywood, USA; 4 Department of Vascular Surgery, Loyola University Medical Center, Maywood, USA

**Keywords:** pulmonary embolism (pe), limb ischemia, patent foramen ovale, embolectomy, venous thromboembolism (vte)

## Abstract

Acute pulmonary embolism (PE) is a manifestation of venous thromboembolic disease with potential serious and life-threatening complications. Management options for acute PE have drastically improved over the last 15 years with the introduction of multidisciplinary pulmonary embolism response teams throughout the world. We present the case of an 18-year-old woman diagnosed with acute PE complicated by near-complete occlusion of her left common femoral artery from a paradoxical embolus in the setting of patent foramen ovale (PFO), managed with surgical pulmonary embolectomy and surgical PFO repair.

## Introduction

Pulmonary embolism (PE) is a dangerous complication of venous thromboembolic (VTE) disease, with manifestations ranging from mild symptoms to death. Approximately 15% of in-hospital deaths in the United States annually have been attributed to acute PE. The most severe complications result from a synergistic effect of obstructive shock, right ventricular (RV) dilation and dysfunction, and hypoxia, ultimately leading to death [1]. Timely evaluation and PE risk stratification of each patient are important as management decisions are made on a case-by-case basis. Low-risk PE manifests with a normal hemodynamic profile and no evidence of RV dysfunction on computed tomography (CT), transthoracic echocardiography (TTE), or biomarker (troponin/BNP [B-natriuretic peptide]) data. Similar to low-risk PE, submassive PE manifests with a normal hemodynamic profile; however, evidence of RV dysfunction on imaging studies and/or on biomarker data is present. Massive PE manifests with systolic blood pressure (SBP) < 90 mmHg or a >40 mmHg decrease in SBP for more than 15 minutes or requiring inotropic support with associated RV dysfunction [2].

In order to better manage acute PE and its complications, hospitals across the world have begun implementing pulmonary embolism response teams (PERT) programs as part of their approach to care. PERTs offer a multidisciplinary approach to PE management by bringing together healthcare providers across different subspecialties in order to rapidly evaluate, diagnose, and ultimately implement treatment strategies for high-risk PE patients [3]. Specific team members vary by institution and may include individuals within the divisions of general and interventional cardiology, vascular and cardiothoracic surgery, pulmonary/critical care, emergency medicine, pharmacy, and hematology [4].

The treatment goal of any PERT program is to facilitate early delivery of safe and effective therapeutic options to patients at high risk of clinical decompensation. The mainstay of therapy relies on early unloading of the RV to reduce RV dilation and to facilitate an early return to physiologic right to left ventricular (RV/LV) ratios, as ratios > 0.9 have been shown to be associated with increased 30-day mortality [5]. The right ventricular outflow tract velocity (RVOT) time integral (VTI) on TTE has also been shown to be a marker of low cardiac output. Normal values are approximately 17 cm, but values < 9.5 cm predict an increased risk of clinical decompensation and PE-related mortality [6]. A range of treatment approaches are available in a physician’s armamentarium and include systemic anticoagulation with unfractionated or low molecular heparin, systemic thrombolysis, and minimally invasive catheter-directed therapies (CDTs) including ultrasound-assisted thrombolysis and aspiration thrombectomy. CDTs have been proven effective while minimizing bleeding risk [7]. For some patients, because of clinical instability, the extent of clot burden, or clinical complexity due to comorbidities, a surgical approach to PE management is offered.

## Case presentation

**Figure 1:**
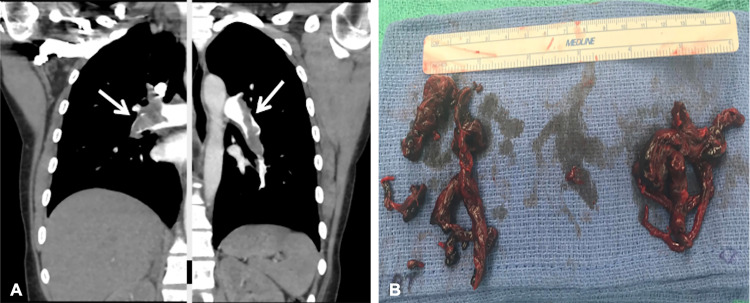
(A) CT pulmonary angiography demonstrating bilateral pulmonary emboli in the main left and right PA (arrows). (B) Thrombi removed during surgical embolectomy. CT computed tomography; PA pulmonary artery

**Figure 2:**
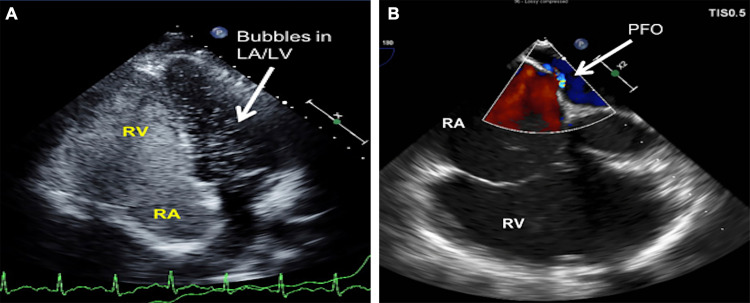
(A) Four-chamber TTE image with agitated saline contrast study demonstrating microbubbles in the LV suggestive of a PFO (arrow). (B) Right-sided focused, intraoperative color Doppler TEE image demonstrating PFO with right-to-left shunt (arrow). LA, left atrium; LV, left ventricle; PFO, patent foramen ovale;RA, right atrium; RV, right ventricle; TEE, transesophageal echocardiogram; TTE, transthoracic echocardiogram

**Figure 3:**
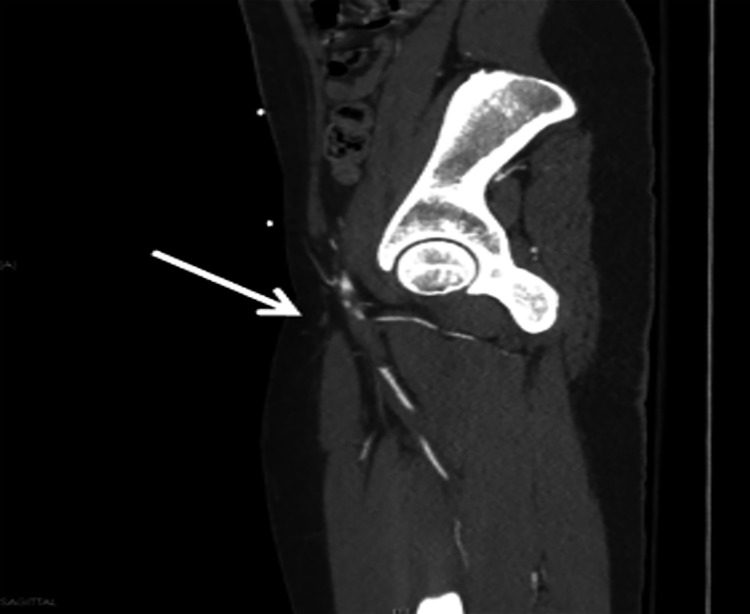
CT angiogram of the left lower extremity demonstrating thrombus with near-complete occlusion of the CFA (arrow). CFA, common femoral artery; CT computed tomography

An 18-year-old woman with preventative oral contraceptive use and recent anterior cruciate ligament repair presented to an outside hospital with shortness of breath, leg pain, and associated loss of consciousness. As part of her workup, she underwent a CT scan of her chest, which revealed acute bilateral PE. Given the complexity of the case, the patient was transferred to our institution for advanced therapies. On arrival, her heart rate was 120 bpm and blood pressure was 112/59 mmHg. Her respiratory rate was 14 breaths/minutes and she was saturating 91% on room air. Initial labs revealed an elevated troponin of 2.47 ng/mL (normal: <0.04 ng/mL), lactic acid of 3 mmol/L (normal: <1.7 mmol/L), and BNP of 174 pg/mL (normal: <100 pg/mL). CT pulmonary angiography was repeated, which demonstrated bilateral PE with a large clot burden in the left and right main pulmonary arteries (PAs) (Figure 1A). Transthoracic echo revealed a RV/LV ratio of 1.53 (normal: <0.9), RVOT VTI of 8 cm (normal: ~17 cm), tricuspid annular plane systolic excursion (TAPSE) of 21 mm (normal: >17 mm), and S’ velocity of 10 cm/s (normal: >9.5 cm/s), suggestive of RV enlargement and dysfunction. Bubble study demonstrated a patent foramen ovale (PFO) (Figures 2A, 2B). Doppler studies of the lower extremities were performed due to decreased pulses, which revealed thrombus in the left common femoral artery with near-total occlusion of flow. A confirmatory CT angiogram of the lower extremities was performed (Figure 3). Given the patient’s complexity, our institutional PERT was activated and immediate collaboration between the divisions of cardiology, cardiothoracic, and vascular surgery was initiated.

## Discussion

Our patient’s presentation was consistent with a submassive PE with an intermediate-high risk of mortality. The initial therapeutic options discussed between the members of the PERT team included systemic anticoagulation with concomitant CDT and simultaneous versus delayed PFO closure or surgical embolectomy with PFO closure at the time of clot retrieval. The timing of PFO closure is important in these patients as the risk of paradoxical embolization must be balanced by the potential acute rise in RV pressure leading to RV failure with early closure. After weighing the patient’s symptoms, overall risk, lack of comorbid conditions, and the high risk of paradoxical embolus leading to a catastrophic stroke with CDT, the decision was made to proceed with surgical embolectomy with simultaneous PFO repair.

Details and techniques of surgical pulmonary embolectomy vary between institutions. Our preference is for bicaval cannulation through median sternotomy using normothermic bypass. Unless there is a clot in transit through a PFO, ischemic arrest is avoided to prevent further injury to the overloaded RV. Caval tapes exclude the right heart to minimize PA flow and to allow closure of the PFO if necessary. A longitudinal arteriotomy in the main PA, with or without extension into the left trunk, and/or directly in the right PA between the greater aortic curvature and the superior vena cava can be created as embolic burden requires. Embolus may be extracted with curved ring clamps. The instrument should not be closed completely, as the embolus may fragment or injury to septations between PA branches may occur. Suction catheters can be advanced into smaller branches to extract embolus, and a milking compression of the lungs may be helpful. Fogarty catheters should not be used because pulmonary anatomy renders them ineffective and dangerous.

The patient underwent a successful bilateral pulmonary embolectomy with surgical PFO closure (Figure 1B). The time from PERT activation to embolectomy was 4 hours. The timing of intervention and a patient’s clinical stability going into the operating room are the main determinants of outcomes after surgical embolectomy. If patients experience cardiopulmonary arrest prior to the operating room, in-hospital mortality is increased substantially compared to similar patients without arrest [8]. On post-operative day 1, the patient underwent left lower extremity arterial embolectomy with patch angioplasty with vascular surgery for the management of her acute limb ischemia. The patient was discharged 14 days post-embolectomy. On her follow-up PERT clinic appointment, her functional status markedly improved with no evidence of post-PE syndrome. Repeat TTE demonstrated recovered RV function. She is currently in her sophomore year of undergraduate studies and doing well.

## Conclusions

While PE is a dangerous cardiovascular condition with the potential for serious complications, the implementation of PERT programs throughout the world over the last decade has drastically changed the landscape of acute PE management via a multidisciplinary approach to care. Through the assessment of various markers of disease severity, such as vital signs, laboratory values, CT obtained RV/LV ratio, and TTE-obtained RVOT VTI, patients can be triaged into the appropriate risk category. This standardized approach to categorization allows for a seamless discussion between members of the PERT team in order to mobilize resources and deliver the best care possible to the patient. In high-risk, clinically stable patients with few or no comorbidities, surgical embolectomy with PFO closure becomes a viable option when the risk of paradoxical embolization is high.
